# Nutrition Behaviour During Pregnancy: Eating Twice as Healthy? A Secondary Analysis of the Nutrition Component of the NELIP Randomized Controlled Trial

**DOI:** 10.3390/nu18152473

**Published:** 2026-07-30

**Authors:** Karishma Hosein, Taniya S. Nagpal, Roberta Bgeginski, Isabelle Giroux, Harry Prapavessis, Michelle F. Mottola

**Affiliations:** 1 R. Samuel McLaughlin Foundation—Exercise and Pregnancy Lab, School of Kinesiology, Faculty of Health Sciences, Western University, London, ON N6A 3K7, Canada; khosein2@uwo.ca (K.H.); rbgegins@uwo.ca (R.B.); 2 Faculty of Kinesiology, Sport and Recreation, University of Alberta, Edmonton, AB T6G 2H9, Canada; tnagpal@ualberta.ca; 3 Children’s Health Research Institute, London, ON N6C 2V5, Canada; hprapave@uwo.ca; 4 School of Nutrition Sciences, Faculty of Health Sciences, University of Ottawa, Ottawa, ON K1N 6N5, Canada; igiroux@uottawa.ca; 5 Exercise and Health Promotion Lab, School of Kinesiology, Faculty of Health Sciences, Western University, London, ON N6A 3K7, Canada; 6 Department of Anatomy & Cell Biology, Schulich School of Medicine & Dentistry, Western University, London, ON N6A 5C1, Canada

**Keywords:** pregnancy, nutrition, eating habits, nutrition intervention, behaviour change, adherence, diet

## Abstract

**Background/Objectives:** To examine adherence to the specific goals of the nutrition component of a healthy lifestyle intervention program during pregnancy (the Nutrition and Exercise Lifestyle Intervention Program; NELIP) from baseline at 12–18 weeks gestation to trial midpoint (24–25 weeks gestation) and endpoint (34–36 weeks gestation). **Methods:** A convenience sample of 90 individuals were included in the secondary analysis. Participants completed the pregnancy intervention in one of three groups: NE, nutrition and exercise introduced simultaneously; N+E, nutrition introduced first followed by exercise introduced at midpoint; or E+N, exercise introduced first followed by nutrition introduced at midpoint. A meal plan with guidelines for carbohydrate and protein consumption was included in the nutrition component, and adherence to the specific meal plan goals was tracked using a novel rubric developed especially for the meal plan in the NELIP intervention. **Results:** All groups had improvements in nutrition adherence from baseline to midpoint, with the NE group scoring highest in adherence (12.9 ± 2.0 points) at midpoint compared to the N+E (11.2 ± 1.8 points) and E+N (11.8 ± 2.0 points) groups (F_(2, 85)_ = 6.08, *p* = 0.00, η^2^ = 0.13). From midpoint to endpoint, N+E was the only group with no decline in adherence. **Conclusions:** The N+E group had the most stable improvements in adherence over the course of the intervention, despite better improvements in adherence from baseline to midpoint in groups NE and E+N. Time of intervention component introduction may have a differential impact on adherence score fluctuation, with baseline nutrition scores potentially influencing nutrition habits over time.

## 1. Introduction

Adherence to a healthy nutrition program during pregnancy can be difficult, and monitoring adherence during an intervention is crucial to ensure that pregnant individuals are meeting the goals of a given nutrition intervention. Some studies do assess adherence to interventions, but they may not be clear about the specialized or individualized nutrition program goals [1,2]. This is particularly important when nutrition is not the only component of the intervention, as additional components, like exercise, will require splitting attentional resources. Measuring adherence over the course of an intervention is also important to get a sense of changes to dietary patterns that occur over the course of pregnancy and to understand which times during an intervention may be more challenging regarding adherence to nutrition goals (i.e., holidays, seasonal changes, specific gestational ages, or adherence changes when new intervention components are introduced).

The present study was undertaken to better understand the complexities and possible nuances of adherence to the nutrition component of an intervention, and to advance the development of the intervention as well as other nutrition behaviour change programs. Previous research in our laboratory [3,4] using the Nutrition and Exercise Lifestyle Intervention Program (NELIP) [5] showed that introducing two behaviour change components (nutrition and exercise) simultaneously or sequentially influence adherence to the intervention. Findings suggested introducing exercise first (at 12–18 weeks gestation) followed by initiating the nutrition component (E+N) at 25 weeks of pregnancy and following both components until delivery showed higher adherence and prevented excessive gestational weight gain than introducing both components simultaneously (NE) or introducing the nutrition component first followed by sequentially introducing exercise (N+E) [4]. The NELIP program has consistently shown positive outcomes like reduced risk of gestational diabetes mellitus (GDM) and excessive gestational weight gain (EGWG) [4,5], and though adherence evaluations have shown favourability to exercise [3], it is known that both exercise and nutrition behaviours are essential for optimal health during pregnancy. In terms of eating habits, pregnant individuals may seek to prioritize improving their nutrition behaviours but often encounter challenges due to factors like self-efficacy for cooking routines, meal prep, changing eating behaviours for one person when food is usually eaten as a family, etc. [6]. In the current study, participants were required to follow a complex meal plan (nutrition component of NELIP) based on a modified gestational diabetic diet (medical nutrition therapy) given to GDM patients [5,7]. We incorporated the GDM meal plan within the NELIP to help prevent excessive gestational weight gain and GDM [4]. We found the lower adherence to the nutrition component compared to the exercise component of NELIP intriguing, which prompted us to investigate the nutrition component more closely, specifically examining adherence to the *specific* goals of the NELIP meal plan to perhaps explain why adherence to the intervention was different between groups (E+N, NE and N+E) [4]. To accomplish this, a nutritional rubric was developed to examine adherence to the finely tuned goals of the meal plan designed specifically for the current study as a deeper secondary analysis of the nutrition component of the NELIP trial.

**Figure 1:**
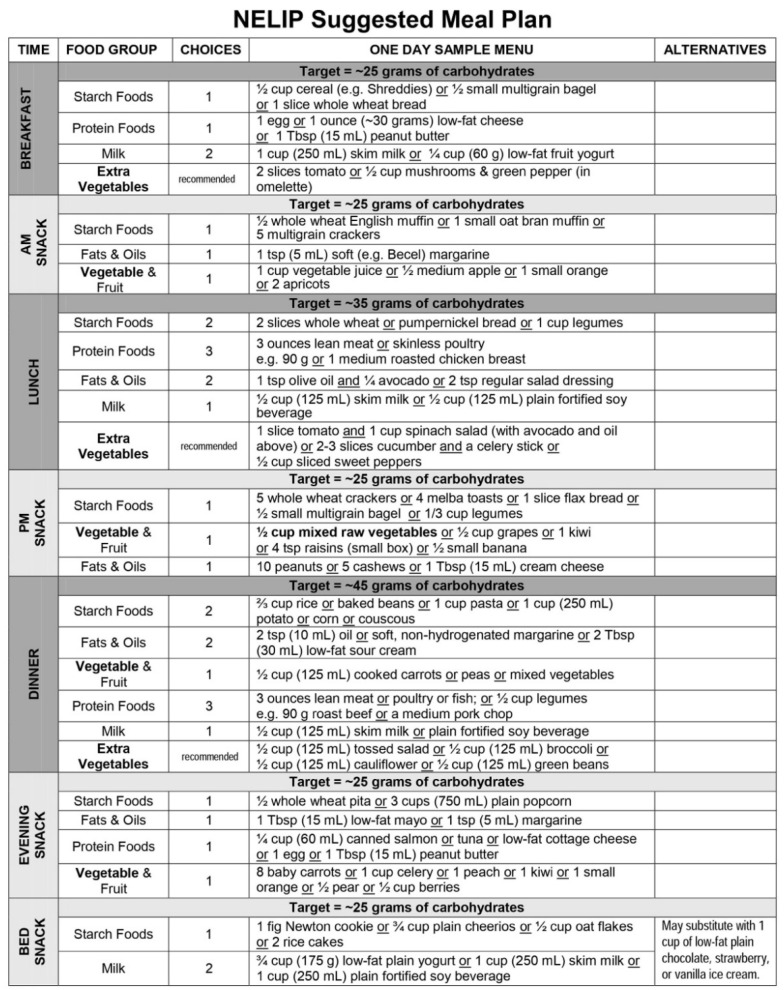

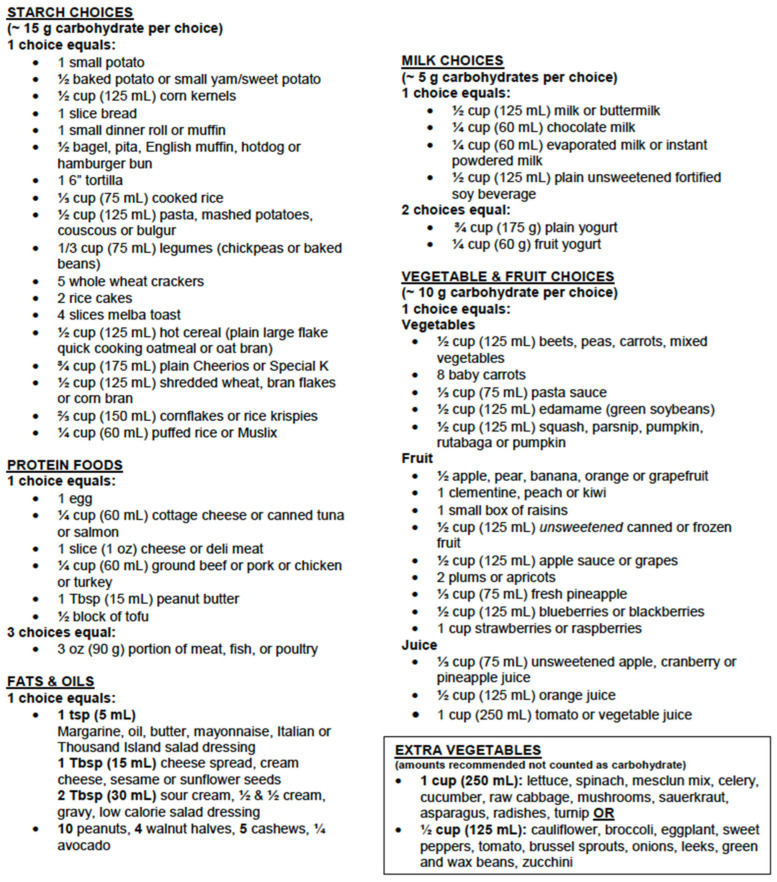
NELIP suggested daily meal plan with specific meal and snack macronutrient distribution and choices.

The modified GDM meal plan (Figure 1) encouraged balanced eating during pregnancy to achieve appropriate gestational weight gain and to prevent the onset of GDM. Specific meal plan goals might be more complex and harder to achieve (decreasing adherence) but might be important to investigate when combined with the potentially easier walking (exercise) behaviour component of the NELIP. The specific goals of the meal plan were: (1) consumption of adequate carbohydrates (CHOs, g) and protein (PRO, g) at *each* meal and snack throughout the day; (2) consumption of three meals and three to four snacks throughout the day; (3) total number of meals and snacks consumed throughout the day; and (4) total number of times a serving of fruits or vegetables were consumed throughout the day. The nutrition component was individualized to the nutritional habits of each participant while they were engaged with the nutrition component of the intervention, which was reinforced during weekly face-to-face meetings at the lab with a member of the research team.

Examining adherence to the specific goals of the GDM meal plan (medical nutrition therapy) is rare in the literature, which prompted us to develop the adherence rubric of the current study (see methods) to examine the nutrition component of the NELIP trial. The newly developed rubric can be utilized to check adherence to nutrition management in GDM patients. The purpose of the present study was to conduct a secondary analysis and to examine more closely the adherence to the NELIP meal plan goals. It was hypothesized that adherence to the specific goals of the nutrition component as scored by the new rubric of the intervention would be higher in those who were introduced to the nutrition program earlier in the pregnancy intervention (at 12–18 weeks gestation) (NE and N+E) compared to those who started later in the intervention (at 24–25 weeks gestation) (E+N), regardless of when the exercise component was introduced.

## 2. Materials and Methods

### Adherence Tool

To understand adherence to the specific goals of the GDM meal plan, a rubric was developed for the current study (Table 1). The rubric represents the first application of the tool and formal validation has not yet been completed. Each item of the rubric was determined based on daily macronutrient consumption frequency that followed the suggestions of the meal plan—three meals and three to four snacks per day. The criteria for meeting each goal of the nutrition component were defined as consuming the appropriate amount of CHOs or protein PRO goals for each meal or snack suggested by the meal plan. Grams of CHOs and PRO consumed were assigned scores using a rating scale from zero to one point. Each level of the rating scale (zero, half point, one point) corresponded to a specific number of grams consumed, as outlined in the rubric. Scores of one point were given if the goal number of grams of CHOs or PRO were consumed, plus one starch “choice” based on the exchange system (outlined in Figure 1) and as suggested by the Diabetes Canada Clinical Practice Guidelines [8], giving a target range (i.e., 25 g + or − 15 g CHOs or 8 g + or − 8 g PRO). A score of 0.5 points was given if the number of grams of CHO or PRO consumed was one “choice” less than the goal number of grams of CHOs or PRO. A score of zero points was assigned for either fewer than the goal grams minus one “choice” or greater than the goal grams plus one “choice” of CHOs or PRO per meal or snack. Additionally, points were awarded for consumption of each recommended meal and snack during the recording day, and for the appropriate total number of meals and snacks consumed. A maximum of 20 points could be achieved, representing perfect adherence to the specific goals of the nutrition component of the intervention.

The current study was part of a larger parallel group three-arm randomized controlled trial in London, Canada, with 1:1:1 allocation ratio, investigating three lifestyle intervention strategies within the NELIP during pregnancy, which was registered with ClinicalTrials.gov (Trial registration number: NCT02804061; Registration date: 14 June 2026). The objective of the larger trial was to evaluate the effectiveness of these three strategies to prevent early or late excessive gestational weight gain and the original sample size required was 81 participants [4]. The RCT protocol followed CONSORT guidelines and was approved by Western University Human Research Ethics Board. Participants were recruited from August 2016 to March 2020 and provided written informed consent before joining the study.

The full study methodology has been previously published elsewhere [3,4,5,9]. Briefly, pregnant individuals were recruited to participate and were excluded from participation if they were >18 weeks pregnant, were <18 years of age, did not have a singleton pregnancy (i.e., no twins or triplets), had existing diabetes, were current smokers, exceeded the physical activity guidelines during pregnancy based on the PARmed-X for Pregnancy [10], had any other chronic diseases or conditions, or had any contraindications to participation in physical activity or exercise. The study had three major visits, at baseline (12–18 weeks gestation) where depending on group each component of the intervention was introduced (nutrition or exercise or both), midpoint (24–25 weeks gestation) where the second component was introduced (exercise or nutrition, with both components continued in all groups until the third visit at endpoint (34–36 weeks gestation).

Baseline data were collected, including maternal age (years), self-reported pre-pregnancy weight (kg), and gestational age (weeks). Current weight (kg) was taken using a calibrated weigh scale, and height (m) was measured using a stadiometer. In the week following the initial visit, participants were instructed to complete, over the course of three consecutive days (two weekdays and one weekend day), a step count log and 3-day food intake record (3dFR). To track step counts, participants were given the option to either use a wrist-worn fitness tracking device or a hip-worn pedometer and were given detailed instructions for use of their chosen step count tracking method. To track food intake, participants were given a food log worksheet to be completed over the 3 recording days. Detailed instructions were given outlining what information to record (time and place of food consumption, method of preparation, portion size, ingredients in compound foods or recipes). Participants were given the choice of completing the 3dFR on the paper worksheet or digitally, in an electronic format. All returned records were examined and confirmed with study participants to identify implausible dietary records and improve data quality. All participants who completed the initial visit were booked to attend a second visit in the lab approximately one week later, at which time participants returned their completed 3dFR and step count logs. Upon return of their records, participants were randomly allocated into one of three intervention strategy groups: NE, nutrition and exercise components were offered simultaneously, introduced at baseline (12–18 weeks gestation) and continued until endpoint (34–36 weeks gestation); N+E, nutrition component of the intervention was introduced at baseline (12–18 weeks gestation) with the exercise component introduced sequentially at midpoint (24–25 weeks gestation), and both intervention components were continued until endpoint; or E+N, exercise component of the intervention was introduced at baseline (12–18 weeks gestation) with the nutrition component introduced sequentially at midpoint and both intervention components were continued until endpoint. All groups were encouraged to follow both components until delivery.

Regardless of intervention strategy group assignment (NE, N+E or E+N), all participants were followed at weekly face-to-face visits in the lab. At these visits, participants were weighed and depending on group allocation were given individualized nutrition counseling and/or participated in a supervised bout of walking exercise. The weekly visits occurred after group assignment from baseline (12–18 weeks gestation) to midpoint (24–25 weeks gestation), and to the endpoint of the intervention (34–36 weeks gestation). Participants repeated the same measures that were performed at study entry (3dFR) at midpoint and at the end of the study. After the midpoint visit, participants were introduced to subsequent additional intervention components, if applicable (i.e., groups N+E and E+N). The same meal plan was used at all time points of the study.

The primary outcome measure of the current study was change in adherence to the specific goals of the nutrition component (meal plan) of the intervention, based on Table 1, at time points 1, 2, and 3 of the study (baseline, midpoint, endpoint).

Since the current study is a secondary analysis of the larger RCT, we used a convenience sample of participants from the NELIP intervention study who completed the 3dFR [4]. Descriptive statistics were calculated for all participant characteristics and for average CHOs (g) consumed at each time point for each group using a two-way ANOVA. Distribution of data was assessed using the Shapiro–Wilk test of normality. Adherence was scored out of 20 points using the developed nutrition rubric for the current study, and an average total score was determined for each intervention group. The nutrition rubric scores were converted to a percentage (%) out of 100 to represent the average adherence scores and to compare to previously reported high adherence scores (70%; [4]). Percent mean adherence was compared across three timepoints during pregnancy using two-way analysis of variance (ANOVA). Repeated measures ANOVA between- and within-groups with post-hoc Bonferroni adjustments, as well as ANCOVA using baseline data as a covariate, were also used. A Chi square analysis was performed to determine if there were significant differences between groups for meeting/not meeting the CHO goals at meals and snacks at each intervention timepoint. All statistical analyses were performed in SPSS (Version 27, SPSS Inc., IBM, Armonk, NY, USA). Results were reported as mean ± standard deviation or n and %. The significance value was set to α = 0.05. Where applicable, effect sizes were calculated using eta-squared (η^2^) to measure proportion of variance.

## 3. Results

**Figure 2:**
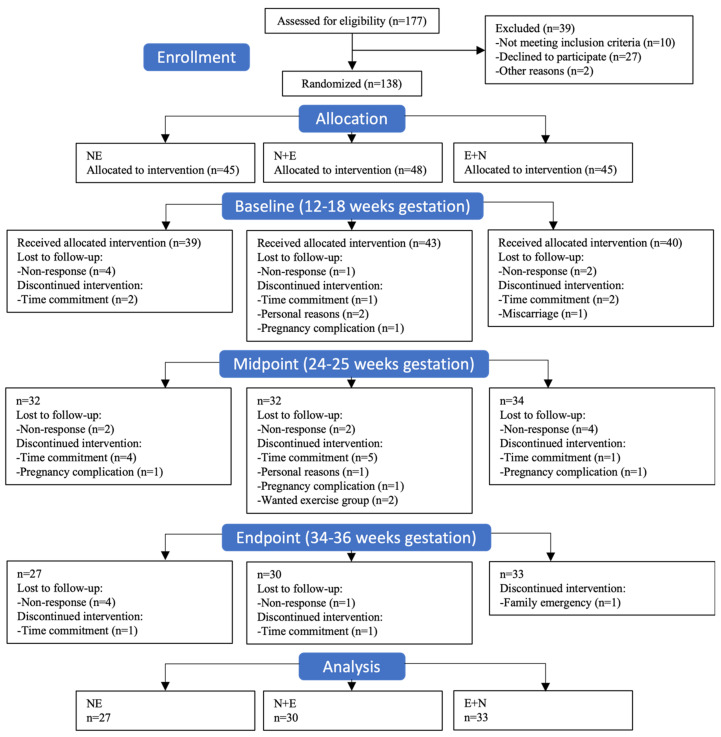
CONSORT flow diagram of the three intervention strategy groups.

Study recruitment ended in March 2020 due to ongoing concerns for vulnerable populations because of COVID-19. Participants enrolled in the RCT who had completed 3dFR assessments at timepoints 1, 2, and 3 were analyzed (n = 90). Stratified by group allotment, the participants to be included for analysis were NE, n = 27; N+E, n = 30; and E+N, n = 33. Participant flow, including reasons for drop-out, is illustrated in Figure 2.

### 3.1. Demographics

Participant demographic characteristics for the current study groups are presented in Table 2. There were no significant differences between the three intervention groups for age, parity, pre-pregnancy BMI (kg/m^2^), gestational age at study entry, ethnicity or education. Over the course of participation in the pregnancy intervention, only one participant developed GDM (information obtained via anecdotal self-report).

### 3.2. Nutrition Rubric Scores

Scores from the nutrition rubric are presented in Table 3. Compared with repeated measures two-way ANOVA, adherence scores between groups showed the score of group NE (11.2 ± 1.6 points) was significantly different (F_(2, 87)_ = 3.95, *p* = 0.02, η^2^ = 0.08) from the score of group E+N (9.0 ± 2.1 points) at baseline. Scores were significantly different (F_(2, 85)_ = 6.08, *p* < 0.001, η^2^ = 0.13) at the study midpoint for group NE (12.9 ± 2.0 points) compared to groups N+E (11.2 ± 1.8 points) and E+N (11.8 ± 2.0 points). Within groups, a significant difference (*p* = 0.04) was observed for the scores at baseline (9.0 ± 2.1 points) and midpoint (11.8 ± 2.0 points) of group E+N. There were differences observed between the groups at baseline and midpoint, with group NE scoring significantly higher on the rubric than group N+E at baseline and higher than both groups N+E and E+N at midpoint. All groups had an improvement in adherence scores from baseline to midpoint. N+E was the only group observed to maintain this score improvement from midpoint to endpoint, as depicted in Table 3. The other two groups, NE and E+N, had decreases in adherence scores from midpoint to endpoint.

### 3.3. Percent Mean Adherence

**Figure 3:**
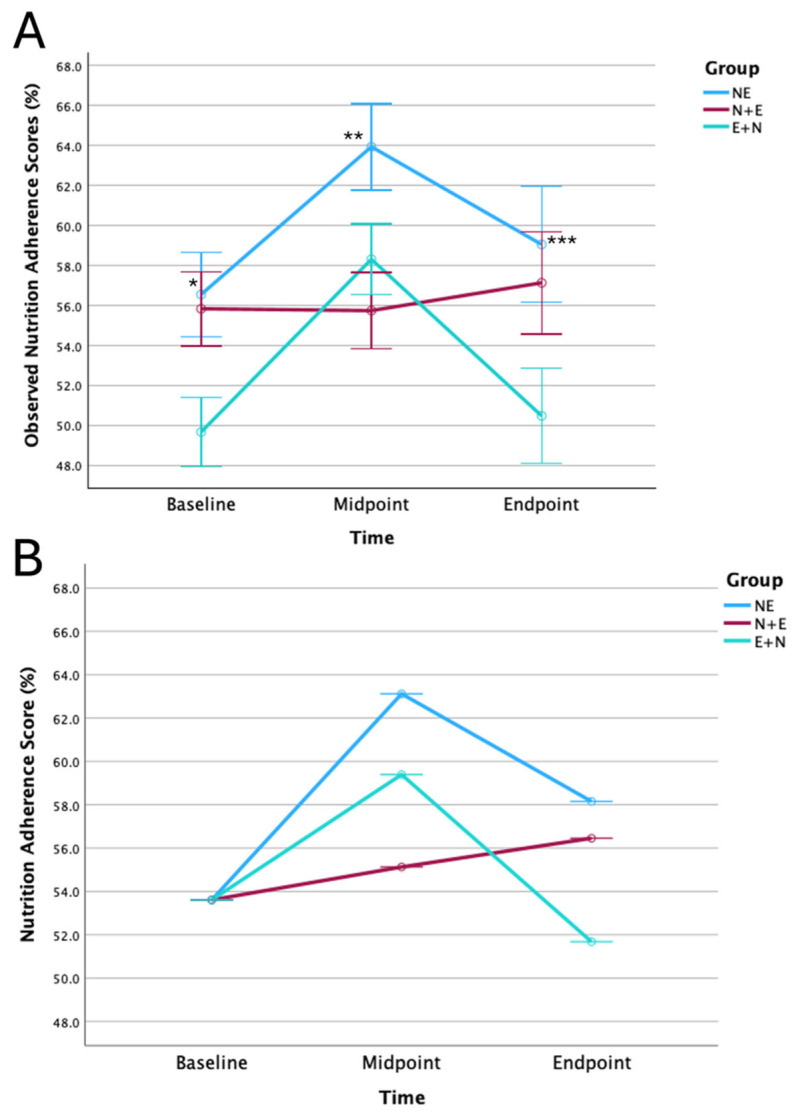
Nutrition adherence scores (%) at baseline (12–18 weeks gestation), midpoint (24–25 weeks gestation), and endpoint (34–36 weeks gestation) during pregnancy. (**A**). Observed nutrition adherence scores (%). (**B**). Nutrition adherence scores with baseline controlled. NE = nutrition and exercise introduced simultaneously, N+E = nutrition introduced first, followed sequentially by exercise, E+N = exercise introduced first, followed sequentially by nutrition. * *p* < 0.05 comparing group NE (56.54 ± 7.8%) to E+N (49.7 ± 11.4%) at baseline. ** *p* < 0.05 comparing group NE (63.9 ± 10.6%) to N+E (55.7 ± 9.3%) at midpoint. *** *p* < 0.05 comparing group NE (59.1 ± 8.9%) to E+N (50.5 ± 17.1%) at endpoint.

For the adherence score percentages (%), a repeated-measures between-subjects ANOVA, presented in Figure 3A, revealed a significant effect of group on adherence score (F_(2,76)_ = 5.46, *p* = 0.006, η^2^ = 0.13), indicating that 12.6% of variance in scores was attributable to group membership. Post-hoc comparisons using Bonferroni adjustment revealed that the NE group scored significantly higher than E+N (*p* = 0.04). A two-way repeated-measures ANOVA conducted within each group showed that NE experienced a statistically significant change in scores over time (F_(2,19)_ = 4.33, *p* = 0.028, η^2^ = 0.31), indicating a large effect. E+N also showed a significant change over time (F_(2,29)_ = 7.80, *p* = 0.002, η^2^ = 0.35), reflecting a large effect. However, N+E did not demonstrate a significant change across time (F_(2,25)_ = 0.16, *p* = 0.85, η^2^ = 0.01), reflecting stability over time. Pairwise comparisons showed that group NE significantly increased in adherence between baseline and midpoint (mean difference (MD) = 7.38, *p* = 0.007). Group N+E showed no significant changes across time, confirming that adherence scores remained stable over all three time points. Group E+N exhibited a significant increase in adherence from baseline to midpoint (MD = 8.63, *p* < 0.001) and a significant decrease in adherence from midpoint to endpoint (MD = −7.82, *p* = 0.024).

A repeated measures analysis of covariance (ANCOVA) was conducted to examine changes in percent adherence to nutrition intervention goals from mid to late pregnancy across the three intervention groups (NE, N+E, E+N), while controlling for baseline adherence (at the time of collecting baseline data, groups had not yet been given nutrition goals to follow). Baseline adherence percentage significantly predicted overall adherence during pregnancy (F_(1,73)_ = 4.18, *p* = 0.044, η^2^p = 0.054), indicating that participants who displayed stronger alignment with adherence to the goals of the nutrition program early in pregnancy, before receiving the meal plan, tended to maintain higher adherence throughout the intervention. These data are presented in Figure 3B. None of the groups achieved 70% adherence to the specific goals of the nutrition rubric.

## 4. Discussion

The current study investigated the nutrition component of the NELIP intervention in more detail than previous work as a secondary analysis by tracking changes in adherence to specific nutrition goals of the intervention meal plan using a novel adherence rubric. Adherence was scored using the meal plan rubric designed for the current study and was defined as meeting the specific dietary intake goals from the meal plan, which included target carbohydrate intakes and timely meals and snacks. The main finding for adherence in this secondary analysis with a modest sample size was that the N+E group, who started the intervention with just the nutrition component with exercise added at 24–25 weeks gestation, had the most stable maintained improvements in adherence in specific meal plan goals over the course of the intervention. The NE and E+N groups had bigger improvements in nutrition behaviour from baseline to midpoint, with their scores increasing by 8.5% and 14%, respectively, compared to just a 1.5% increase in the N+E group. Interestingly, from baseline when the nutrition component had not yet been introduced to midpoint when it was given, the exercise-first group’s eating habits were already in line with the specific goals of the nutrition intervention, despite not yet receiving the meal plan. However, when given the meal plan at midpoint, the E+N group’s adherence to the nutrition goals decreased from midpoint to endpoint. These results suggest that time had a differential impact on adherence score fluctuation depending on the intervention group. When baseline nutrition scores were controlled, eating habits before the start of the intervention may influence nutrition habits over time, especially when participants are given a specific meal plan that may be close to their existing eating habits.

One important factor to consider is that irrespective of treatment group allocation, adherence scores on the meal plan rubric ranged between 50 and 64%. This is much lower than our previous work of overall general adherence scores to both the nutrition and exercise components [3,4]. However, the current study provides some support for our rationale that diving deeper into the more complex nutrition behaviour (i.e., GDM meal plan) is more challenging and therefore more difficult in adherence. This suggests that when complex meal plans are initiated into an intervention trial, whether offering each component simultaneously or sequentially, perhaps alternative behaviour change strategies need to be implemented for more complex nutrition meal plan interventions to increase adherence to ≥70%. This may also be true in GDM management where a complex meal plan and activity program is initiated as part of the treatment plan for GDM. Nevertheless, even with lower adherence to the specific goals of the meal plan, overall general adherence goals to both the nutrition and exercise components were met by most of the participants (NE = 62.1 ± 18.5%; N+E = 69.8 ± 13.5%; E+N = 76.5 ± 16.3%) [4], preventing excessive pregnancy weight gain and GDM. More research is necessary to tease out more complex issues relating to GDM meal plan adherence.

Participation in exercise has previously been linked to influencing other health-conscious behaviours, like improvements in healthy eating. A study of women with pre-pregnancy overweight or obesity that participated in an exercise intervention until 36 weeks of pregnancy also increased consumption of nutrient-dense foods in the same time frame, compared to overweight/obesity and normal weight groups that did not exercise [12], suggesting a link between participation in exercise during pregnancy and dietary improvements. In the current study, the N+E group had a modest improvement in adherence from midpoint to endpoint while both the NE and E+N groups had decreases in their meal plan nutrition goal adherence, dropping 8% and 5%, respectively, potentially indicating that the N+E group was able to maintain and improve adherence to the nutrition goals as pregnancy progressed. The N+E group began the intervention with just the nutrition component, and had the exercise component added at midpoint, suggesting that timing of the introduction of a nutrition component as early as possible during pregnancy before adding an exercise component may be key to adopting lasting improvements to complex eating habits. However, there may also be a confounding effect of exercise on nutrition habits, in line with the findings of Bgeginski et al. [4] and Nagpal et al. [3], suggesting that exercise introduced first may be a potential impetus or “gateway” for improvements to nutrition behaviour. In the current study, the nutrition first followed by exercise group (N+E) had the best percent adherence (64%) to the specific goals of the nutrition intervention but not the overall program (including exercise), which reached 70% [4]. Perhaps the specific goals of the meal plan were more difficult to achieve but the broad goals of the nutrition and exercise components were more attainable. This distinction is important to consider, as previous work [3,4] did not use the same specific nutrition goals as the present study and instead focused on the broad goals of total carbohydrate intake, calorie intake, and submission of a weekly food intake log. Future work with these strategies could consider both the specific and broad goals of the nutrition and exercise components of the intervention to better understand the relationship between these behaviours and the unique challenges that exist with adherence. However, regardless of the percent adherence to specific goals of the meal plan not quite reaching 70% in all of the groups, when all components were taken together, as mentioned above, all groups prevented excessive pregnancy weight gain and gestational diabetes mellitus [4].

It is known that as pregnancy progresses it may become more difficult to adhere to specific nutrition recommendations [13], regardless of when dietary guidelines or nutrition recommendations are introduced. In a cross-sectional analysis of 572 pregnant individuals, De Pasquale et al. [13] found that more than 50% did not adhere to dietary recommendations. Emerging research has highlighted that behavioural interventions in pregnancy are often skewed by selection bias as individuals who have a higher income or education are more likely to participate [14]. Consequently, critical social factors that implicate nutrition adherence are overlooked, such as food insecurity, variability in cultural practices, and socioeconomic status, which is also related to education levels [15,16,17,18]. In fact, a systematic review found that higher education levels, like in the current study population, were a principal predictor of adherence to specific nutrition goals [19]. This could be leveraged for nutrition intervention design to develop specific goal recommendations that can be communicated in a clear and accessible way to pregnant individuals with different levels of education, as maternal education continues to be a strong determinant of dietary patterns [20,21]. Indeed, in a longitudinal study using food frequency questionnaires and food diaries to understand maternal and paternal dietary patterns during pregnancy and at 4 years postpartum, “health conscious” patterns of eating had the strongest associations with the mother’s socioeconomic background, including higher educational attainment and older maternal age [20]. The same study [20] also found that having a BMI in the overweight category before pregnancy had a lower association with the “health conscious” eating pattern, which is in line with other work [13,19,22,23], suggesting that pre-pregnancy BMI as a predictor of healthy eating adherence should be taken into consideration along with the eating pattern and education level of the mother and family at the start of the intervention. Of note though, BMI should not be used as a sole indicator of adherence as this contributes to weight stigmatizing assumptions, such as pregnant individuals with larger bodies eat poorly, as this in and of itself can be a barrier to participation in prenatal health behaviour change programs [24,25].

To our knowledge, this is the first study to develop and use a nutrition rubric designed for the specific goals of a modified GDM meal plan used in the nutrition component of a lifestyle intervention during pregnancy (NELIP). The strength of this rubric is in its versatility because it can be adapted for several scenarios, including pregnant individuals who may need support to manage gestational weight gain, who are very physically active and have different dietary requirements, or who begin pregnancy with other complications. The versatility of the rubric is a factor of its design, which considered the Diabetes Canada Clinical Practice Guidelines [8], or recommended carbohydrate consumption (grams) and determined ranges for point scoring. In addition, using this rubric as a baseline assessment shows the initial eating habits of the participants and how closely they aligned with the meal plan before the start of an intervention, which may potentially influence the adherence to the nutrition component as pregnancy progresses.

Another major strength of the current study was the assessment of food intake and macronutrient composition at multiple timepoints during pregnancy and the intervention, which worked to give a comprehensive understanding of how, when advised to control carbohydrate intake, changes to eating habits were actually being implemented by participants. Additionally, employing the use of a prospective food intake record removed the risk of recall bias. Participants were given weekly, face-to-face individualized nutrition education and support, which have been cited as important factors to improve motivation to making healthy behaviour changes during pregnancy [26].

A limitation of the current study was the sample size, which was a convenience sample of 90 participants taken from the NELIP RCT, resulting in a different number of participants included in each of the three intervention groups (27 vs. 30 vs. 33). As a secondary analysis, the current sample was not adequately powered, since the sample size calculations suggested 129 participants (approximately 43 per group) were necessary to detect differences for the primary outcome in the current study of adherence to the specific goals of the nutrition component (meal plan) of the NELIP intervention. Effect sizes were reported to aid in interpretation of the data, but it should be noted that the reduced sample size could impact the robustness of the observed findings and the possibility of a type II error.

This was the first application of the nutrition adherence rubric, which was developed based on the previous adherence tool [9] and will be used in subsequent future applications. Future work plans to assess the reproducibility and validity of the nutrition adherence rubric tool. Other limitations included rounding of numbers for scoring, which could have over- or under-estimated macronutrient intake. Additionally, the 3dFR may not have been the most representative of how participants were eating, as it only measured three days (two weekdays and one weekend day). Weekly records were not included in analysis but were used as a tool during the intervention for motivation, counseling, and to stay on track with the intervention goals. Eating habits recorded in the 3dFRs may have been affected by participants’ education levels (including health and nutritional literacy), occupations (i.e., shift work), food security, having other children at home for whom to prepare meals, the place and time that food is being consumed, or not having control over the food shopping or preparation. There was also risk of social desirability bias where participants reported altered nutrition behaviours to reflect what is expected of them, and bias due to the use of self-report measurement tools, which are commonly used in studies assessing nutrition behaviours There were several behavioural factors that were not measured in the current study, including baseline food literacy, household food security, intrinsic motivation, self-efficacy, or readiness for dietary change. Indeed, more research is needed to elucidate the factors that may be influencing nutrition adherence outcomes, and particular focus should be applied to unique barriers and facilitators to lifestyle behaviour change that may come about when nutrition and exercise are combined in complex intervention strategies. Further investigation into the psychological state of participants during and after an intervention is also necessary, as a large part of behaviour change action may be intangible factors that must be measured from a qualitative behaviour change theory lens to better understand intrinsic motivations, capabilities, impulses, perceptions, and intentions related to habit formation, behaviour change, and adherence differences between intervention groups.

## 5. Conclusions

Adhering to the specific goals of a nutrition intervention during pregnancy, such as a modified GDM meal plan, is challenging. The results of the current study, observed with a relatively modest sample size using a secondary analysis design, showed that introduction of a healthy eating plan early in the second trimester of pregnancy may be most effective at influencing nutrition behaviour changes for the duration of pregnancy and improving eating habits long-term. Delivering a nutrition component first followed by exercise led to the most consistent adherence to specific goals, which may be an important factor to consider for future work concerning influencing lasting behaviour change, which may be important for those who are at risk for GDM or who need management of the disease. However, eating habits (at baseline) coming into the intervention (before beginning to follow the meal plan) may also influence nutrition habits over time, especially when participants are given specific meal plan goals. Support for meeting macronutrient intake goals should be increased as pregnancy progresses, especially for breakfast meals. Future studies should aim to utilize intervention-specific adherence tools, like a nutrition rubric, to track if participants are meeting specific nutrition goals and to be used as a guide for individualized feedback. Additionally, examining participants’ understanding of nutrition goals, as well as the barriers (like low food literacy and food insecurity) and facilitators (like social support) of nutrition behaviour adherence, could help the integration of strategies to improve eating behaviours during pregnancy and the maintenance of developed nutrition habits into the postpartum period. It is critical to examine adherence to specific nutrition goals during pregnancy but also postpartum, as children tend to follow similar patterns of eating as their parents [20], so maintaining healthy nutrition habits developed during pregnancy may be key to ensuring the health of several generations.

## Figures and Tables

**Table 1:** Adherence scoring tool based on the specific goals of the meal plan of the nutrition component of the three intervention strategies, developed for the current study.

Points		0	0.5	1
CHO intake (g)	Breakfast (25 g)	<10 or >40 g	10–24 g	25–40 g
	+1 PRO (8 g)	0 or >16	1–7 g	8–16 g
	Morning Snack (25 g)	<10 or >40 g	10–24 g	25–40 g
	Lunch (35 g)	<20 or >50	20–34 g	35–50 g
	+3 PRO (24 g)	<16 or >32 g	16–23 g	24–32 g
	Afternoon Snack (25 g)	<10 or >40 g	10–24 g	25–40 g
	Dinner (45 g)	<30 or >60 g	30–44 g	45–60 g
	+3 PRO (24 g)	<16 or >32 g	16–23 g	24–32 g
	Evening Snack (25 g)	<10 or >40 g	10–24 g	25–40 g
	Bedtime Snack (25 g)	<10 or >40 g	10–24 g	25–40 g
	+1 PRO (8 g)	0 or >16	1–7 g	8–16 g
1st meal		Did not eat		Ate
2nd meal		Did not eat		Ate
3rd meal		Did not eat		Ate
1st snack		Did not eat		Ate
2nd snack		Did not eat		Ate
3rd snack		Did not eat		Ate
4th snack		Did not eat		Ate
Meals consumed (n per 24 hr)		<1 or >3	2	3
Snacks consumed (n per 24 hr)		0 or >4	1–2	3–4

CHO = carbohydrate, PRO = protein, g = grams, n = number, hr = hours.

**Table 2:** Participant demographic characteristics of the convenience sample at study entry by group allocation. All data shown as mean ± standard deviation unless otherwise indicated.

	NE (n = 27)	N+E (n = 30)	E+N (n = 33)
Age (years)	32.7 ± 4.2	32.1 ± 3.6	32.5 ± 3.1
Parity (n; %)			
0	18; 66.7	19; 63.3	25; 75.8
1	5; 18.5	10; 33.3	7; 21.2
2	4; 14.8	1; 3.3	1; 3
Pre-pregnancy BMI (kg/m^2^)	26.3 ± 4.9	25.7 ± 4.7	26.0 ± 5.3
Pre-pregnancy BMI category (n; %)			
Normal weight	12; 44	14; 47	20; 61
Overweight	11; 41	12; 40	8; 24
Obese	4; 15	4; 13	5; 15
Gestational age at study entry (weeks)	15.3 ± 2.2	15.1 ± 2.3	14.8 ± 2.5
Ethnicity (n; %)			
Caucasian	22; 81	27; 90	31; 94
Asian	2; 7	0; 0	1; 3
Hispanic	1; 4	1; 3	0; 0
African American	0; 0	0; 0	1; 3
Mixed ethnicity	2; 7	2; 6	0; 0
Education (n; %)			
High school	1; 4	0; 0	0; 0
College	1; 4	5; 17	3; 9
Bachelors	9; 33	13; 43	16; 48
Masters	14; 52	8; 27	7; 21
Doctorate	1; 4	1; 3	5; 15
Professional degree (MD, JD, teacher’s	1; 4	3; 10	2; 6
college, veterinary, accounting)			

NE = nutrition and exercise introduced simultaneously, N+E = nutrition introduced first, followed sequentially by exercise, E+N = exercise introduced first, followed sequentially by nutrition. BMI = body mass index. MD = medical doctor. JD = juris doctor (law degree).

**Table 3:** Adherence to the specific goals of the nutrition component rubric scores of each group at major study visits. Scores are shown as mean ± standard deviation.

Group	Baseline (12–18 Weeks)	Midpoint (24–25 Weeks)	Endpoint (34–36 Weeks)
NE	11.2 ± 1.6 ^+^ (n = 27)	12.9 ± 2.0 * (n = 25)	11.9 ± 1.8 (n = 22)
N+E	10.9 ± 1.9 (n = 30)	11.2 ± 1.8 (n = 30)	11.4 ± 2.2 (n = 27)
E+N	9.0 ± 2.1 ^++^ (n = 33)	11.8 ± 2.0 (n = 33)	10.8 ± 2.2 (n = 29)
Effect size	**0.08**	**0.13**	0.04

NE = nutrition and exercise introduced simultaneously, N+E = nutrition introduced first, followed sequentially by exercise, E+N = exercise introduced first, followed sequentially by nutrition. * *p* < 0.05 comparing group NE to group N+E and group E+N at midpoint. ^+^ *p* < 0.05 comparing group NE to group E+N at baseline. ^++^ *p* < 0.05 comparing group E+N at baseline to midpoint. Large and medium effect sizes at each timepoint are indicated in bold referring to Cohen’s (1988) [11] criteria: Partial eta squared for One-Way ANOVA: small = 0.01, medium = 0.06, large = 0.14.

## Data Availability

The original contributions presented in this study are included in the article. Further inquiries can be directed to the corresponding author.
